# Down syndrome cell adhesion molecule like-1 (DSCAML1) links the GABA system and seizure susceptibility

**DOI:** 10.1186/s40478-020-01082-6

**Published:** 2020-11-30

**Authors:** Yoneko Hayase, Shigeru Amano, Koichi Hashizume, Takashi Tominaga, Hiroyuki Miyamoto, Yukie Kanno, Yukiko Ueno-Inoue, Takayoshi Inoue, Mayumi Yamada, Shigehiro Ogata, Shabeesh Balan, Ken Hayashi, Yoshiki Miura, Kentaro Tokudome, Yukihiro Ohno, Takuma Nishijo, Toshihiko Momiyama, Yuchio Yanagawa, Akiko Takizawa, Tomoji Mashimo, Tadao Serikawa, Akihiro Sekine, Eiji Nakagawa, Eri Takeshita, Takeo Yoshikawa, Chikako Waga, Ken Inoue, Yu-ichi Goto, Yoichi Nabeshima, Nobuo Ihara, Kazuhiro Yamakawa, Shinichiro Taya, Mikio Hoshino

**Affiliations:** 1 Department of Biochemistry and Cellular Biology, National Institute of Neuroscience, National Center of Neurology and Psychiatry (NCNP), 4-1-1 Ogawa-higashi, Kodaira, Tokyo 187-8502 Japan; 2 Graduate School of Medicine Faculty of Health Science, Department of Laboratory Medicine, Kyoto University, Kyoto, 606-8501 Japan; 3 Laboratory for Neural Circuit System, Institute of Neuroscience, Tokushima Bunri University, Sanuki, 769-2300 Japan; 4 International Research Center for Neurointelligence (IRCN), The University of Tokyo, Tokyo, 187-8502 Japan; 5 Research Center for Dynamic Living Systems, Graduate School of Biostudies, Kyoto University, Kyoto, 606-8501 Japan; 6 Laboratory for Molecular Psychiatry, RIKEN Center for Brain Science, 2-1 Hirosawa, Wako, Saitama 351-0198 Japan; 7 Department of Pharmacology, Osaka University of Pharmaceutical Sciences, Takatsuki, Osaka 569-1094 Japan; 8 Department of Pharmacology, Jikei University School of Medicine, Tokyo, 105-8461 Japan; 9 Genetic and Behavioral Neuroscience, Gunma University Graduate School of Medicine, Maebashi, Gunma 371-8511 Japan; 10 Institute of Laboratory Animals, Graduate School of Medicine, Kyoto University, Kyoto, 606-8501 Japan; 11 Laboratory Animal Research Center, Institute of Medical Science, The University of Tokyo, Tokyo, 108-839 Japan; 12 Omics-Based Medicine, Center for Preventive Medical Science, Chiba University, Chiba, 260-0856 Japan; 13 Department of Pediatric Neurology, National Center Hospital, NCNP, Tokyo, 187-8551 Japan; 14 Department of Mental Retardation and Birth Defect Research, NCNP, Tokyo, 187-8551 Japan; 15 Foundation for Biomedical Research and Innovation, Kobe, 650-0047 Japan; 16 Graduate School of Medical Science, Nagoya City University, Nagoya, 467-8601 Japan

**Keywords:** DSCAML1, Seizure susceptibility, Ihara epileptic rat, Gabaergic neurons, Patient-type model mouse

## Abstract

The Ihara epileptic rat (IER) is a mutant model with limbic-like seizures whose pathology and causative gene remain elusive. In this report, via linkage analysis, we identified Down syndrome cell adhesion molecule-like 1(*Dscaml1*) as the responsible gene for IER. A single base mutation in *Dscaml1* causes abnormal splicing, leading to lack of DSCAML1. IERs have enhanced seizure susceptibility and accelerated kindling establishment. Furthermore, GABAergic neurons are severely reduced in the entorhinal cortex (ECx) of these animals. Voltage-sensitive dye imaging that directly presents the excitation status of brain slices revealed abnormally persistent excitability in IER ECx. This suggests that reduced GABAergic neurons may cause weak sustained entorhinal cortex activations, leading to natural kindling via the perforant path that could cause dentate gyrus hypertrophy and epileptogenesis. Furthermore, we identified a single nucleotide substitution in a human epilepsy that would result in one amino acid change in DSCAML1 (A2105T mutation). The mutant DSCAML1^A2105T^ protein is not presented on the cell surface, losing its homophilic cell adhesion ability. We generated knock-in mice (*Dscaml1*^*A2105T*^) carrying the corresponding mutation and observed reduced GABAergic neurons in the ECx as well as spike-and-wave electrocorticogram. We conclude that DSCAML1 is required for GABAergic neuron placement in the ECx and suppression of seizure susceptibility in rodents. Our findings suggest that mutations in DSCAML1 may affect seizure susceptibility in humans.

**Electronic supplementary material:**

The online version of this article (10.1186/s40478-020-01082-6) contains supplementary material, which is available to authorized users.

## Introduction

Recent advances in human genetics have identified a number of genes associated with epilepsy [1, 2]. Although many scientists have been analyzing these genes, the machinery underlying epileptogenesis remains elusive. Furthermore, there are many patients with seizures that are refractory to anticonvulsants, highlighting the need to develop new treatments for epilepsy.

The Ihara epileptic rat (IER) is a rat mutant line with limbic-like seizures that has been used as an animal model for temporal lobe epilepsy in humans [3]. Abnormal behaviors such as hyperactivity with frequent mastication, erratic running, jumping or “wet-dog” shaking occur from the age of two months. Spontaneous generalized tonic and clonic convulsions (GTCs) become evident at the age of 5–6-months-old (M). The frequency and the degree of seizures increase with aging (Additional file 1: Video 1) [3, 4] Neuropathological investigation revealed that IERs exhibit small neuronal disarrangements consisting of irregular arrangements of pyramidal cells, gap formation in the array of the pyramidal cell layer, and abnormal tiny loci of neuronal clusters in the hippocampal formation (i.e., microdysgenesis; MDG) [5]. In addition, IERs exhibit behavioral impairment in social interaction, controlling emotions and spatial learning even before the onset of any seizure-related behaviors [6]. Similarly, some cases of human epilepsy are often accompanied by such psychiatric symptoms. Therefore, IER represents a good animal model for human epilepsy with intellectual disabilities. However, the underlying molecular pathology nor the responsible gene for IER has been clarified.

DSCAML1 (Down Syndrome Cell Adhesion Molecule Like 1) is a transmembrane protein with ten immunoglobulin (Ig) and six fibronectin type III domains [7], whose structure is similar to its related family member, DSCAM. DSCAML1 in human chromosome (Chr) 11 and DSCAM in Chr 21 are neuronal cell adhesion molecules working on mutual repulsion after cell attachment. In fruit flies and mammals, DSCAM has been shown to participate in neuronal tiling, avoidance of neurite fasciculation, as well as axon guidance [8, 9]. Similarly, DSCAML1 has been shown to be involved in neuronal tiling and avoidance of neurite fasciculation in the mouse retina [10] and dendrite formation in in vitro hippocampal culture [11].

In this study, we analyzed the pathology of the IERs and identified the responsible gene (*Dscaml1*). We found that GABAergic neurons were severely reduced in the ECx, where the excitability was abnormally enhanced. Furthermore, we performed genomic sequencing for the *DSCAML1* gene in epilepsy patients with accompanying intellectual disabilities, and identified one nucleotide replacement that results in a one amino acid change from alanine to threonine at the 2105th residue of DSCAML1 (DSCAML1^A2105T^). We generated *Dscaml1*^*A2105T*^ knock-in mice by genome editing and observed abnormalities similar to those found in IER, raising the possibility that *Dscaml1* is related to human seizure susceptibility.

## Materials and methods

Animals: The rat and mouse strains, genotype detecting primers, and vendors used in this study are listed in Additional file 2: Table 1. The sources of the materials are also listed in Additional file 2: Table 1. All animal experiments were quantified double-blinded. Anesthesia in general experiments was performed with a mixture of Medetomidine/Midazolam/Butorphanol tartrate (0.75/4/5 mg/kg for mouse and 0.15/2/2.5 mg/kg for rat, Fujifilm-WAKO), and that in physiological experiments was performed with 1–2% isoflurane inhalation (Fujifilm-WAKO). Lidocaine (Xylocaine_jelly_®, Aspen Japan) was added as a local anesthetic for craniotomy.

The *GAD67*^*GFP*^ knock-in mouse strain [12] was crossed to *WT*, *Dscaml1*^*GT2*^ and *Dscaml1*^*A2105T*^ mouse, respectively, for identifying GABAergic neurons.

Physiological studies: Rat EEG [3, 4], mouse Electrocorticogram (EcoG) [13], rat amygdala kindling experiments [14] and inhibitory post-synaptic current (IPSC) recording methods [15] were performed as described previously. Spike-and-wave discharges were defined by at least three consecutive negative spikes, 6–10 Hz in frequency, and with spike amplitude over 300% of baseline activity at wakefulness. Spike-and-wave discharges were scored manually and quantified for 6 h during the light phase (9:00−15:00). We performed all voltage-sensitive dye (VSD) imaging of the rat brain slices according to protocols approved by the Animal Care and Use Committee of Tokushima-Bunri University. Brain slices (400 µm thick) prepared from wild type (WT) rats and IERs were used for VSD imaging as described previously [16–18].

**Fig. 1:**
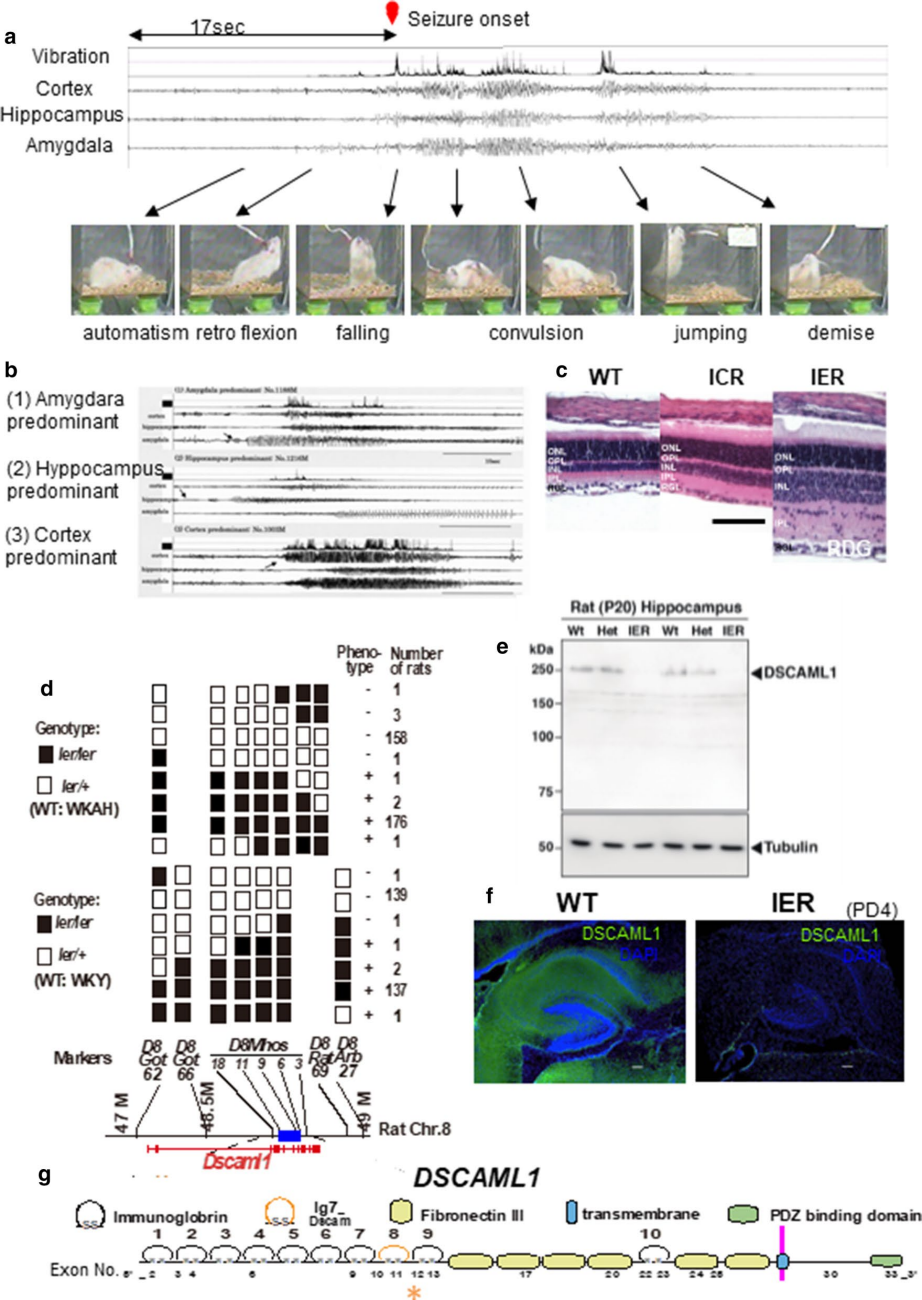
Physiological features of IER and linkage analysis *of Ier*. **a**, **b** Representative EEG (**a**, **b**) and seizure sequences (**a**). The seizures were originated 7/12 in the amygdala (1), 4/12 in the hippocampus (2), and 1/12 in the cortex (3) (*n* = 12, 10 M IERs). Arrows indicate the initiation of each seizure in the EEG of **b**. **c** HE staining of retinal sections of indicated genotypes at PD20. *ONL* Outer nuclear layer, *OPL* outer plexiform layer, *INL* inner nuclear layer, *IPL* inner plexiform layer, *RGL* Retinal ganglion cell layer. **d** Mapping of *Ier*. (WKAH × IER) F1 × IER backcross and (WKY × IER) F1 × IER backcross used to genetic linkage and haplotype analysis. Marks represent rats exhibiting (+) and not exhibiting (−) retinal dysgenesis (RDG) and hippocampal microdysgenesis (MDG), respectively. White boxes represent homozygosity for the *Ier* allele, while black boxes highlight heterozygosity for the wild type (WKAH/WKY) allele. The genome region spanning D8Mhos11 and D8Mhos6 was identified as the responsible genomic region for *Ier*, which is highlighted by the blue bar. **e** Immunoblot analysis to estimate DSCAML1 protein expression in the hippocampus of WT, heterozygous (Het) and homozygous (IER) rats at PD20. **f** Immunohistochemical staining of DSCAML1 in the hippocampus of PD20 wild type littermates and IER. Nuclei are visualized with DAPI. **g** Schematic picture for DSCAML1 protein. Asterisk indicates the position of the one base change in IER

Linkage analysis: IERs have congenital morphological abnormalities: the retinal dysgenesis (RDG; Fig. 1c) and the neuronal microdysgenesis in the hippocampus (MDG). Using these phenotypes with simple sequence length polymorphism (SSLP) markers [19, 20], we performed linkage analysis to detect the responsible genomic region for IER (Fig. 1d). For the linkage analysis, rats were randomly allocated to experimental groups. Then, female Wistar-Kyoto rats (WKY: WT strain) or female WKA-Hokkaido rats (WKAH: WT strain) and male IERs were crossed to obtain the F_1_ progenies, respectively. Each F_1_ female and IER male were then mated to obtain backcrossed progeny. A total of 343 WKY-backcrossed rats and 282 WKAH-backcrossed rats were obtained. The eyes and brains of all animals were histologically examined for the RDG and the MDG at PD50, using 5 µm-sliced paraffin embedded sections with Hematoxylin–eosin (Muto) stains. The modes of inheritance for the RDG and the MDG were investigated by analyzing the phenotypes in each group: IER, F_1_, WKY and WKAH backcross progenies. The length of each PCR product of the SSLP marker of the backcross rats was compared by the gel electrophoresis. Electrophoresis was performed on a 4% NuSieve3:1 agarose gel (Lonza) at 120 V for 80 min. The SSLP primers used were produced in the previous studies [19]. 92 polymorphic markers in the intervals of 20 centi-Morgan (cM), approximately, on each chromosome were used as described previously [20]. The responsible gene for RDG and MDG was located close to the late-onset-cataract (*Cati1*) locus previously mapped on rat Chr 8. The polymorphic markers between *Thy1* and *D8Rat40* on rat Chr 8 were used in this study were listed in Additional file 2: Table 1. Genomic DNA was extracted from the tails of IER, WKY, WKHA, F_1_ and backcrossed progeny (PX-80, Kurabo Co. Ltd.), and PCR amplified. The PCR conditions were as follows; the reaction volume was 25 µm, 50% GoTaq Master Mix and Polymerase (Promega), using 35 cycles of PCR with a 50–55 °C annealing temperature. MapManager computer program was used for the linkage analyses [21].

**Fig. 2:**
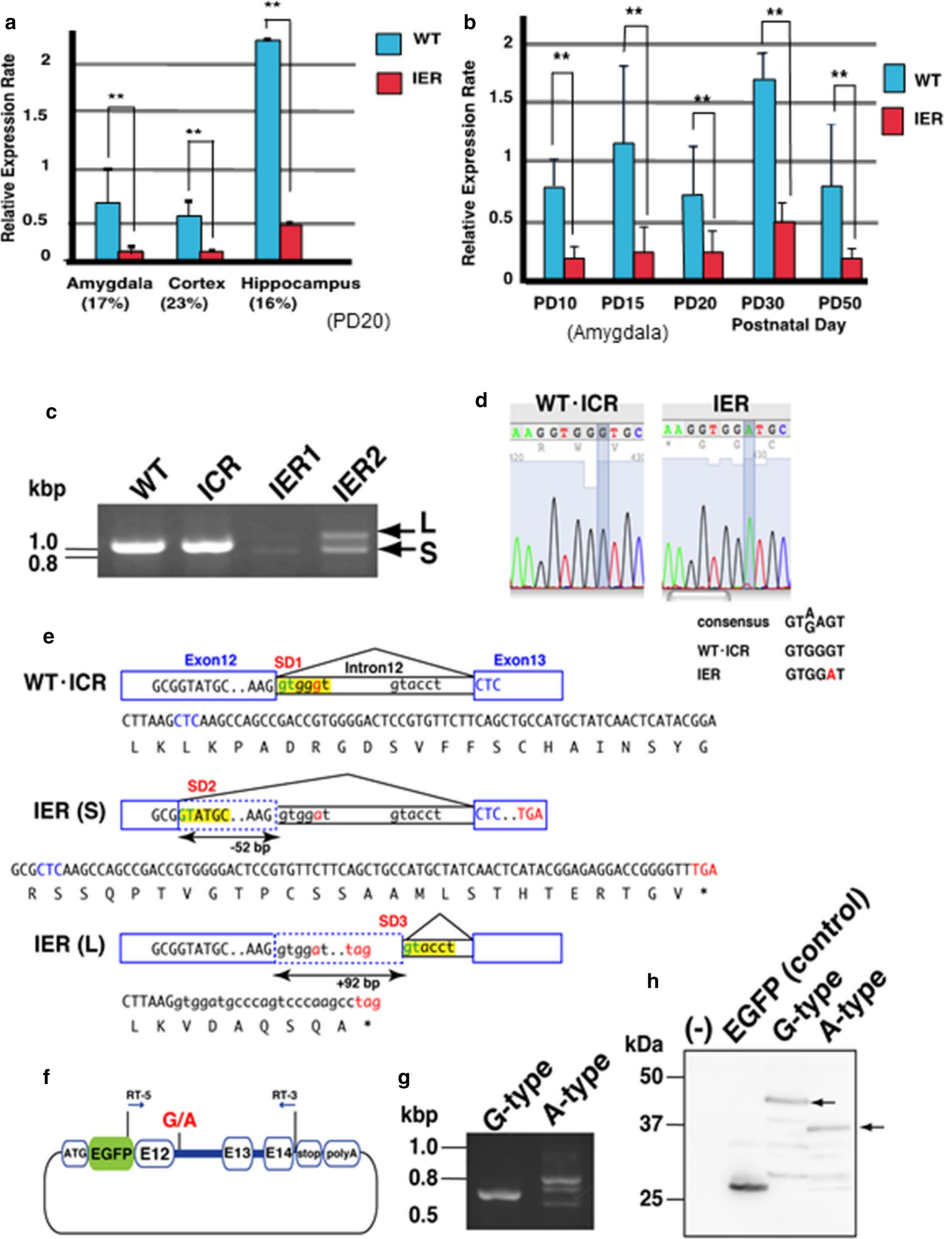
Abnormal splicing of *Dscaml1* in IER. **a** Relative expression levels of the *Dscaml1* transcripts in indicated brain regions of WT and IER at PD20 measured by quantitative RT-PCR analyses (*n* = 6 per genotype, **p < 0.01, Student’s *t-*test, Error bar: s.e.m.). **b** Temporal expression profiles of *Dscaml1* transcripts in rat amygdala at indicated postnatal stages (*n* = 6 per genotype, **p < 0.01, Student’s *t-*test, Error bar: s.e.m.). **c** RT-PCR to amplify *Dscam1* cDNA spanning from exons 9 to 14 of PD20 hippocampus of indicated genotypes. **d** One base change of IER genome with showing the alignments of the consensus sequences for the SD1 splicing donor site. **e** Schematic picture showing the abnormal splicing between exons 12 and 13 in IER. Asterisks indicate translational stop codons that appear in both shorter (S) and longer (L) transcripts. **f** Schematic for the G-and A-type expression vectors for in vitro splicing assay. **g** Expression of G- and A-type *Dscaml1* in COS7 cells transfected with *pEGFP-Dscaml1* (exon 12–14). **h** Immunoblot analysis with an anti-GFP antibody detected the EGFP-DSCAML1 fusion proteins. Arrows indicate signals at predicted sizes

In vitro splicing assay: The genomic regions ranged from exon 12 to 14 (containing exon 12, intron 12, exon 13, intron 13 and exon 14) of *Dscaml1* in IER or ICR were amplified by PCR, then inserted in pEGFP-C1 (Thermo Fisher). COS7 cells were transfected with pEGFP-*Dscaml1* (exons 12–14) using Lipofectamine 3000 reagent (Thermo Fisher). Transfected cells were lysed and extracted total proteins were subjected to SDS-PAGE, followed by immunoblot with anti-GFP antibody (Fig. 2h).

*Dscaml1* quantification: To confirm expression levels of the *Dscaml1* in rats or in mice brains, we performed quantitative RT-PCR analysis using FAM™ dye-labeled TaqMan Gene expression assay for rat (Rn01421925_mH) and for mouse (Mm01174247_m1) (Thermo Fisher). RNA from each brain (hippocampus and amygdala, respectively) were extracted by RNeasy Mini kit (Qiagen). Reverse transcription was performed by ReverTra Ace qPCR RT Kit (TOYOBO). *Dscaml1* transcripts were detected with ABI 7300 (Thermo Fisher) from total cDNA samples.

*DSCAML1* genomic sequencing with genomic DNA purified from patient blood samples: The blood samples of the families were obtained from the “Depository of the patients with epilepsy and intellectual disability” of the NCNP from the approved project “Research and utilization of bio bank of hereditary developmental disorder” [22, 23]. We performed genomic sequencing for *DSCAML1* by using genomic DNA purified from the blood samples. There were no cases of DSCAML1^A2105T^ substitution in the control 60 patients (30 males and 30 females) who had only intellectual disability without epilepsy.

Mouse genome editing: *Dscaml*^*A2105T*^ (human *DSCAML1* p.2105T A > T, c.6307 G > A) knock-in mouse were generated via zygote electroporation utilizing the CRISPR/Cas9 genome editing system [24]. We designed the CRISPR RNA (crRNA) in exon 33 to target the coding sequences near p.2105T (Fig. 6d). The crRNA and the trans-activating crRNA (tracrRNA) were chemically synthesized (FASMAC). A single-strand DNA (ssDNA) containing the *c.6307 G* > *A* substitution was chemically synthesized (Eurofins) and used as the repair template. Cas9 protein (50 ng/µl, Guide-it Recombinant Cas9 Nuclease, Clontech), crRNA (100 ng/µl), tracrRNA (100 ng/µl), and ssDNA (500 ng/µl) were introduced into *B6C3F*_*1*_ (SLC) fertilized eggs by electroporation: 30 V (3 ms ON + 97 ms OFF) by 7 square pulses (BEX Genome Editor GEB15). The electroporation zygotes were transferred into the oviducts of pseudo-pregnant *B6C3F*_*1*_ (SLC) female to obtain knock-in founders. In order to evaluate the substitution, the target region of the PCR-amplified tail DNA was cut with *NarI* restriction endonuclease (NEB) (Fig. 6d), and also confirmed by the Sanger sequencing. The *Dscaml1*^*A2105T*^ KI mouse proband was used in experiment by backcrossing with *WT* (*C57BL/6N* (SLC)) for 7 generations and was confirmed that there were no abnormalities in the 3 predicted off-target sites by Sanger sequence. Primers used in detection for off-target sites are described in Additional file 2: Table 1.

Histological studies: Methods for hippocampal primary neuron culture, immunohistochemistry, and Western blot analyses were described in our papers [25–27]. The antibodies used in this study are listed in Additional file 2: Table 1. The neurons' fluorescence-images were incorporated into Neurolucida software (Mbf) and Image J to perform the single neuron tracing and measure the dendrite length. The antibodies used in this study were listed in Additional file 2: Table 1. We raised rabbit anti-DSCAML1 polyclonal antibody against GST-DSCAML1 (231–478 amino acids) fusion protein.

Generation of DSCAML1-expressing stable L cells: L fibroblasts were grown in DMEM containing 10% fetal bovine serum (FBS) in an air–5% CO_2_ atmosphere at constant humidity. To obtain L cells stably expressing EGFP only, DSCAML1^WT^ with EGFP, DSCAML1^WT^ with mCherry, or DSCAML1^A2105T^ with EGFP, L cells were transfected with pCAG-EGFP, pCAG-DSCAML1^WT^-ires-EGFP, pCAG-DSCAML1^WT^-ires-mCherry, or pCAG-DSCAML1^A2105T^-ires-EGFP with pSVIISRa vector containing the neomycin resistance gene using Lipofectamine3000 (Thermo Fisher) as described in the manufacturer’s instructions, and neomycin-resistant clones were selected. DSCAML1-expressing stable cells were grown in DMEM containing 10% FBS and 100 µg/ml of G418.

Cell Aggregation Assay: Aggregation assay was carried out according to previous reports [28, 29]. With minor modifications. Cells were collected in PBS containing 0.05% Trypsin and 1 mM EDTA, and centrifuged at 1000 rpm for 5 min. A suspension of single cells (3 × 10^5^ cells/500 µl) was prepared in DMEM medium. 250 µl aliquots of DSCAML1^WT^ expressing cells (red) were co-cultured with 250 µl aliquots of EGFP only, DSCAML1^WT^, or DSCAML1^A2105T^ expressing cells (green), respectively, in 1.5 ml tubes. Cell mixtures were rotated on the mini disc rotor (Bio-craft) at 37 °C for 60 min. Mixed cells were placed on coverslips and then clustered cells were counted.

Statistical analysis: In culture studies, the numbers of cells used for each calculation are more than 50, and the values shown are means ± s.e.m. of triplicates (Student’s *t-*test; *p < 0.05, **p < 0.01, ***p < 0.001). In the ECoG experiments, spike-and-wave discharges were scored manually and quantified for six hours during the light phase (9:00 − 15:00). The values shown means ± s.e.m. of *n* = 5–7 indicated genotype in the figure (Man-Whitney test; *p < 0.05, **p < 0.01, ***p < 0.001). For other in vivo studies, the numbers of rats or mice of indicated genotypes used for each calculation are described in the figure legends, and the values shown are means ± s.e.m. of triplicates.

## Results

### *Dscaml1* is the responsible gene for IER

IERs exhibit hereditary spontaneous late-onset epilepsy (Additional file 1: Video 1). EEG measurements were performed with a 10 month-old (M) IER (Fig. 1a) using a seizure monitor [4]. The initial spike was observed frequently in the amygdala, sometimes in the hippocampus and in some cases in the cerebral cortex (Fig. 1b). As mentioned previously, we concluded that IER is a good model for limbic-like seizures [3].

We attempted to find the causative gene for this mutant rat using linkage analysis by focusing on morphological features of IER in the hippocampus (i.e., microdysgenesis, MDG) [3, 5], and in the retina (i.e., retinal dysgenesis, RDG) (Fig. 1c), both of which were observed as early as PD15, the pre-epilepsy stage. We identified a causative locus in the *D8Mhos6* to *D8Mho11* genomic region (blue line in Fig. 1d). According to the NCBI rat genome database (NC005107.4 Chr8 Rnor_6.0), the *Dscaml1* gene is the only known gene located in this responsible region for IER. In IERs, the *Dscaml1* transcripts and protein were significantly reduced in various brain regions including amygdala, hippocampus and cortex at all developmental stages (Figs. 1e, f, 2a, b).

We subsequently performed sequencing on genomic DNA of ICR (the original strain of IER [30]) and IER within the region corresponding to the causative locus that we identified in IER. We identified a single-nucleotide change (c.2733 + 5G > A (NM_001108141.1)) in the splicing donor site of exon 12 (Figs. 1g, 2d). This mutation seems to disrupt the consensus splicing donor sequence. We detected abnormal splicing patterns of *Dscaml1* transcripts composed of very weak shorter (S) and longer (L) extra bands in IERs (Fig. 2c), by RT-PCR that amplified exons 9–14 of *Dscaml1* on hippocampal transcripts. The S and L bands used abnormal splicing donors (SD2 and SD3 in Fig. 2e, respectively), which caused frame-shifts that generated premature stop codons. To test whether this single nucleotide change from G to A caused abnormal splicing in IER, we constructed two expression vectors (G-type and A-type) which contained EGFP cDNA followed by *Dscaml1* genomic DNA between exon 12 and exon 14 (Fig. 2f). Abnormal splicing variants basically identical to those in IER were detected only in A-type transfected COS7 cells, which were confirmed by DNA sequencing (Fig. 2g). We detected EGFP-fusion proteins that matched the size of normal (G-type) and short (A-type) ones of the expected size by premature stop codon usage (Fig. 2h). These findings suggest that this nucleotide change results in abnormal splicing and loss of functional DSCAML1 protein in IER.

**Fig. 3:**
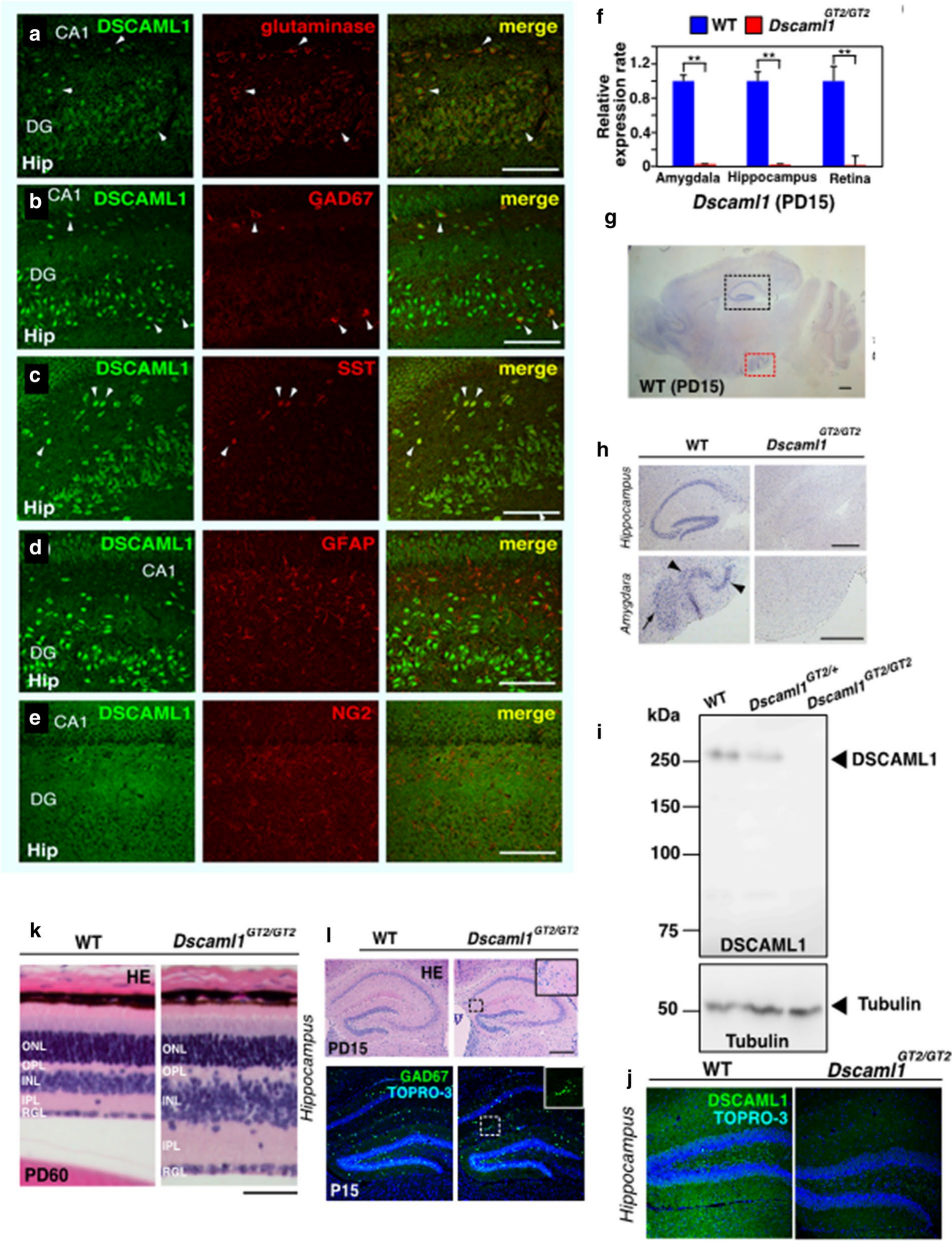
*DSCAML1*^*GT2/GT2*^ mice exhibit IER-like phenotypes. **a–e** Double immunostaining with DSCAML1 (green) and specific markers (red), such as glutaminase (**a**), GAD67 (**b**), SST (**c**), GFAP (**d**) and NG2 (**e**) to the rat hippocampus at PD20. Arrowheads indicate colocalization of DSCAML1 and markers. Scale bars: 100 µm. **f** Quantitative RT-PCR to *Dscaml1* transcripts in indicated regions of *Dscaml1*^*GT2/GT2*^ mice at PD15 (*n* = 10 per genotype; Student’s *t-*test; **p < 0.01, Error bar: s.e.m). **g** In situ hybridization of sagittal sections of wild type mouse brains probed with *Dscaml1* at PD15. Scale bar: 500 µm. **h** High magnified pictures of the areas of rectangles areas of **g** and pictures of the corresponding areas of *Dscaml1*^*GT2/GT2*^ mouse. Arrowheads and an arrow indicate *Dscaml1* expression in the hippocampus and amygdala, respectively. Scale bars: 500 µm. **i** Immunoblot analyses of lysates from the PD15 hippocampus with anti-DSCAML1 and Tubulin antibodies. **j** Distribution of DSCAML1 protein in the mouse hippocampus at PD15. Nuclei were counterstained with TOPRO-3. **k**, **l** Retinal (PD60, **k**), and hippocampal (PD15, **l**) structures visualized with HE staining. Inset in **l** is magnified rectangles to display abnormal cell clustering in the hippocampus of *Dscaml1*^*GT2/GT2*^ mouse. Scale bars: 100 µm (**k**), 200 µm (**l**)

In mice and rats, we observed that *Dscaml1* transcripts and protein were broadly expressed in the brain of WT (Fig. 3a–e, g), partly consistent with a previous report [7]. Double immunostaining with DSCAML1 and other markers revealed that DSCAML1 is expressed in most of the neuronal cells in the hippocampus (Fig. 3a–c), the cortex and the amygdala (data not shown) of wild type rats, including both excitatory and inhibitory neurons, as well as somatostatin (SST) neurons. However, DSCAML1 was barely detected in astrocytes (GFAP) and very faintly in oligodendrocytes (NG2) (Fig. 3d, e). A similar expression profile was observed in wild type hippocampal cultures of rats and mice (data not shown).

We obtained a loss of function allele for *Dscaml1* gene in mice (TIGM), *Dscaml1*^*GT2*^, which contained a gene trap vector in the third intron of the *Dscaml1* gene. We confirmed that *Dscaml1* transcripts and protein were hardly detected in the amygdala, hippocampus, and retina of the homozygous mice (*Dscaml1*^*GT2/GT2*^) (Fig. 3f–j). We also observed RDG (Fig. 3k) and MDG (Fig. 3l) in *Dscaml1*^*GT2/GT2*^ mice. From these findings, we concluded that *Dscaml1* is the responsible gene for IER.

### GABAergic dysfunction in IER

**Fig. 4:**
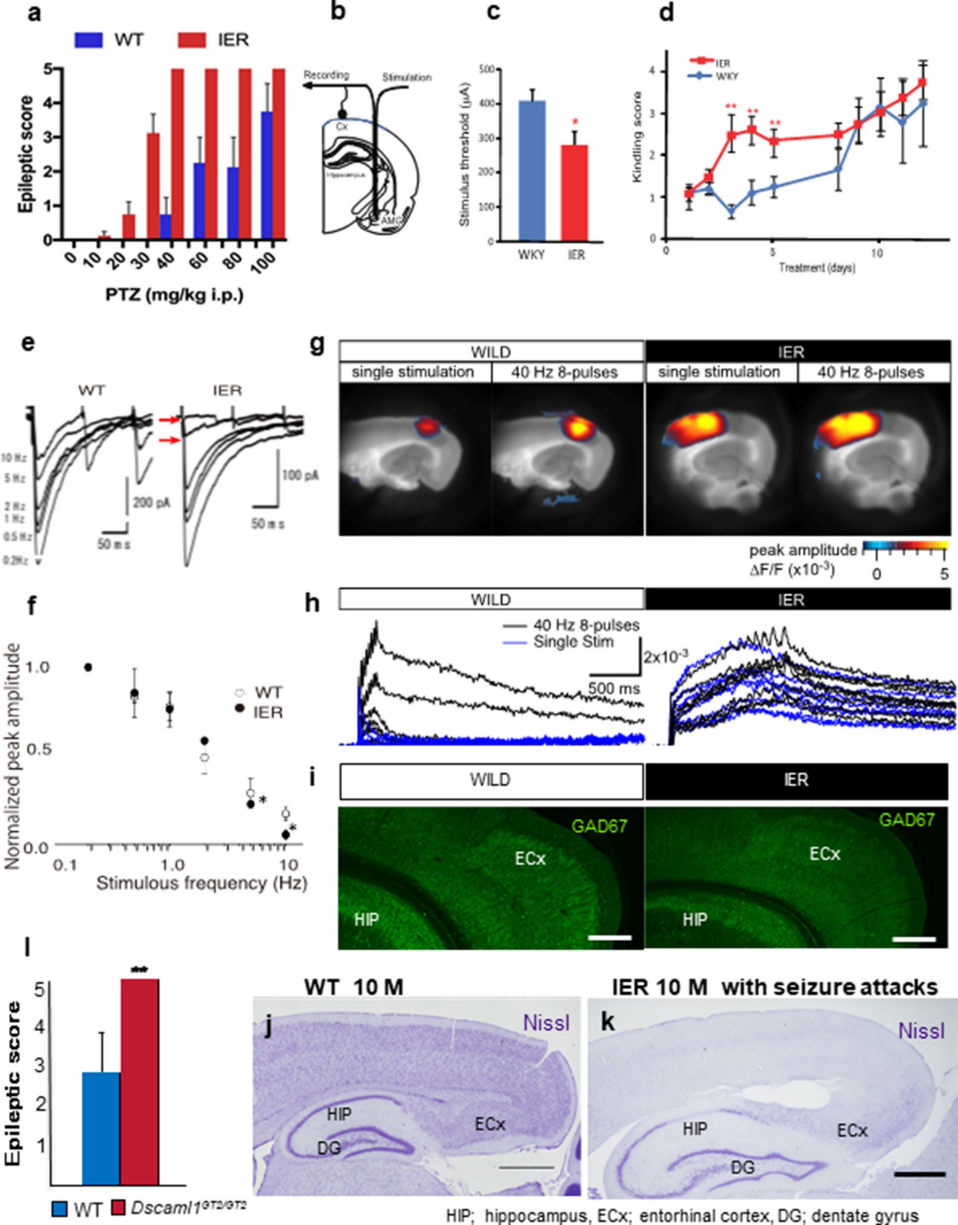
Epileptic susceptibility of the IER and sustained cortical activation evoked from the ECx of IER. **a** PTZ administration to wild type rats and IER at PD20 at indicated doses. The epileptic score based on Racine's score is indicated (*n* = 6 F_6, 98_ = 5.691, *p* < 0.001, 2-way ANOVA). **b** Schematics picture for electrical amygdala kindling. **c** Stimulus threshold for after-discharge at the amygdala on the first day (*n* = 8 per genotype, *p < 0.05, Student’s *t*-test, Error bars: s.e.m.). **d** Kindling score based on Racine's score at each day of the experiment (*n* = 8 per genotype, **p < 0.01, Student’s *t*-test, Error bars: s.e.m.). **e** Examples of IPSCs evoked at various stimulus frequencies (0.2–10 Hz) from pyramidal neurons in the basolateral amygdala of WT and IER. IPSCs showed further frequency-dependent suppression at 5–10 Hz in IER (red arrows). **f** The relationship between normalized IPSC peak amplitude and stimulus frequency. Frequency dependent IPSC impairment was prominent more than 5 Hz stimulations in IER. At the low frequency stimulation (0.2–2 Hz), IPSC is maintained in both WT and IER (*n* = 8 per genotype, *p < 0.05, Student’s *t*-test, Error bars: s.e.m.). **g**, **h** Representative cortical activation upon electrical stimulation with VSD-imaging in the indicated phenotypes (single or 40 Hz eight pulses); VSD amplitude maps (**g**) and time courses (**h**). **i** Distribution of inhibitory (GAD67-positive) neurons in the ECx of WT and IER. Scale bars: 500 µm. **j, k** Nissl staining of the ECx of WT (**j**) and IER (**k**) at 10 M. IERs were experienced frequent seizures. Scale bar: 1 mm. **l** PTZ administration to wild type mice and *Dscaml1*^*GT2/GT2*^ mice at 40 mg/kg. The epileptic score based on Racine's score is indicated (*n* = 4 per genotype; Student’s *t-*test; **p < 0.01, Error bar: s.e.m)

Epilepsy is a disorder in which the balance between cerebral excitability and inhibition tipped toward uncontrolled excitability [31]. We examined the seizure susceptibilities of young IERs and the *Dscaml1*^*GT2/GT2*^ mice at the stage before seizure onset (PD20) by exposing them to the GABA_A_ receptor antagonist, pentylenetetrazol (PTZ). At all doses tested, IERs (Fig. 4a) and *Dscaml1*^*GT2/GT2*^ mice (Fig. 4l) exhibited much stronger Racine’s epileptic scores [32], compared to control. Next, we conducted amygdala electrical kindling stimulation to IER at a pre-onset stage (PD50) (Fig. 4b). The stimulation threshold of after-discharge of IERs on the first day was significantly reduced (Fig. 4c). Moreover, the generalized seizures due to focal stimulation of amygdala were greatly facilitated in IERs compared to controls (Fig. 4d). These findings suggest that the neuronal network in the limbic area was abnormally susceptible to excitatory stimuli in IER, even at pre-onset stages.

**Fig. 5:**
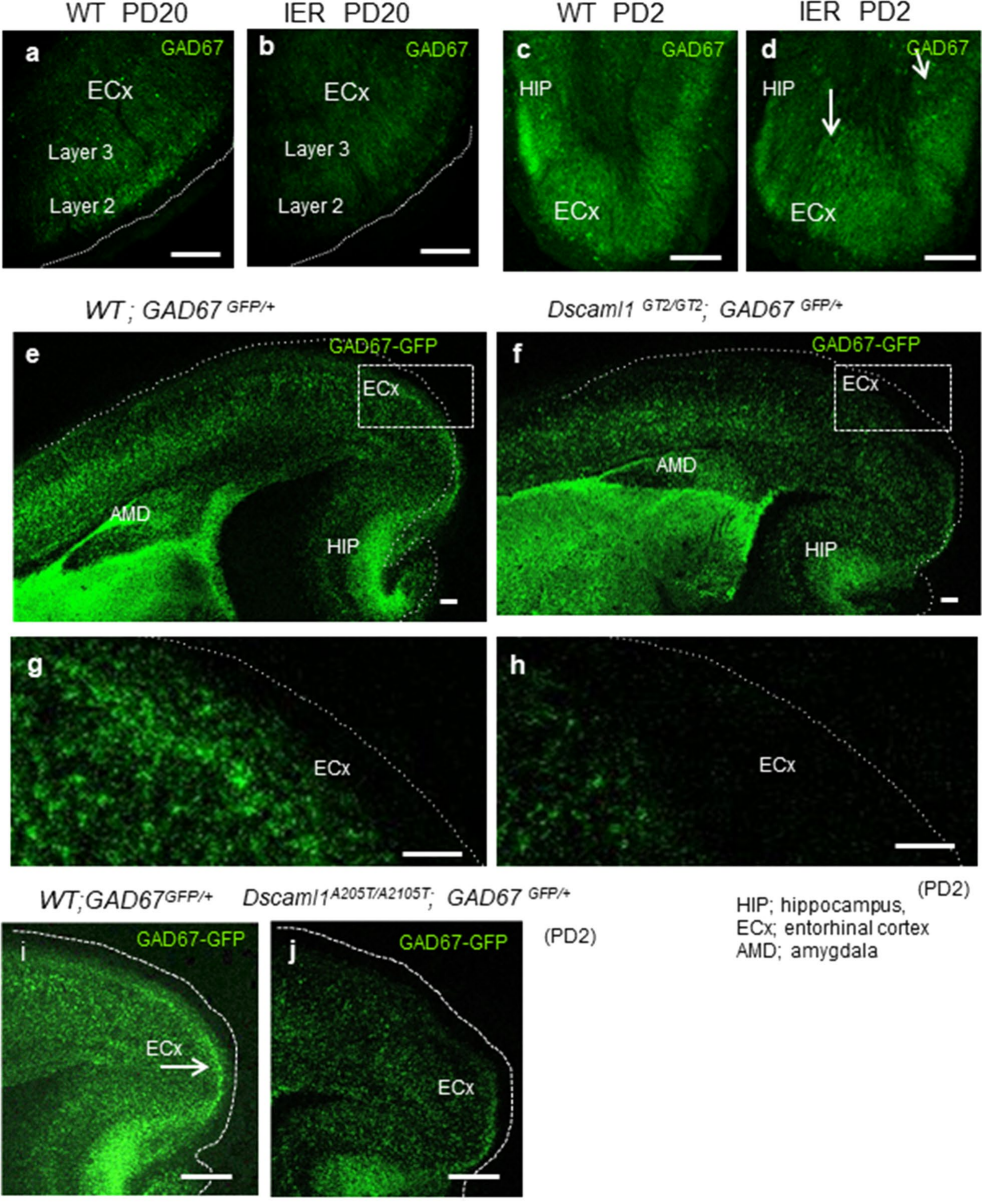
GABA neurons in the ECx of *DSCAML1*^*GT2/GT2*^ and *DSCAML1*^*A2105T2/A2105T*^ mice. **a–d** Distribution of inhibitory (GAD67-positive) neurons in the ECx of the indicated genotypes at PD20 (**a**, **b**) and PD2 (**c**, **d**). Arrows indicate small clusters of GABAergic neurons in IER. **e–j** Morphological changes of inhibitory neurons due to DSCAML1 deficiency. Photo of the limbic area of the distribution of GFP-positive (GAD67-positive) neurons in *WT*; *GAD67*^*GFP/*+^ mouse (**e**, **g**) and *Dscaml1*^*GT2/GT2*^; *GAD67*^*GFP/*+^ mouse (**f**, **h**) in the lateral ECx at PD2 of each indicated genotypes. Scale bars: 100 µm (**e**, **f**), 1000 µm (**g, h**). **i, j** GABA neurons in the lateral ECx are decreased in *Dscaml1*^*A2105T*^ mouse at PD2. Scale bars: 100 µm

We already presented the abnormal neuron clustering, observed in the IER hippocampus of IER as MDG [5]. The small clusters that consist of GABA neurons were seen often in IER hippocampus and amygdala even before the seizure onset (PD 2) (Fig. 5d). Similar clustering of inhibitory neurons was also observed in the hippocampus and amygdala of *Dscaml1*^*GT2/GT2*^*; GAD67*^*GFP/*+^ mice (Fig. 3l). It is known that DSCAML1 is involved in tiling of certain types of neurons in the retina by presumable homo-repulsive effect [10]. Therefore, the clustering of GABA neurons in IER and *Dscaml1*^*GT2/GT2*^ may be caused by the loss of cell-repulsing effects of DSCAML1 protein.

Next, we performed electrophysiological analysis in WT and IER amygdala. At low frequency stimulation, features of IPSCs in pyramidal cells of amygdala slices (amplitude, slope, etc.) were not significantly different between IER and wild type littermates. However, frequency-dependent suppression of IPSCs during sustained stimulation, which was normally observed in wild type littermates, was greatly increased in IERs (arrows and asterisks in Fig. 4e, f). This points to a malfunctioning of the inhibitory neuron system in the IER amygdala. Although we have not clarified the underlying pathological machinery, one possible explanation can be that the impaired electrical feature was caused by abnormal distribution of inhibitory neurons in the amygdala.

In IERs*,* it seems that the inhibitory system is mildly impaired in the hippocampus and amygdala from the pre-epileptic stage. However, we were unsure whether severe seizures observed in IERs were caused only by such minor abnormalities in these areas. As epileptic seizure worsens, IERs showed remarkable wavy dentate gyrus expansion (Fig. 4j, k) and mossy fiber sprouting in the hippocampus [33], but not hippocampal sclerosis. Marked changes in dentate gyrus in response to seizure progression suggested a substantial impact on IER epilepsy of the perforant path that projects from the entorhinal cortex (ECx) [34, 35].

To test the activities of the ECx, we estimated neuronal excitation in brain slices containing the ECx and the hippocampus at postnatal-week (PW) 20, prior to seizure onset, with the voltage-sensitive dye (VSD) imaging method [16–18] (Fig. 4g, h; consecutive images were not shown). First, electrical stimulation was given to the surface layers of the lateral ECx, and the spread of the neuronal excitation was recorded. As evident in Fig. 4g, h, both a single pulse stimulus and a 40 Hz 8-pulse stimulus induced extensive activation in the IER cortex while they evoked smaller and limited activation in the wild type. The depolarizing responses were longer in IER than in wild type especially in the single stimulus experiment. The 40 Hz stimulations induced the oscillatory responses in IER, which were barely observed in the wild type. The potential activity was also weak in the hippocampus and amygdala of both IER and WT during stimulation.

Interestingly, GAD67 immunostaining revealed that GABA neurons were severely reduced in layer II of ECx of IER at PW20 (Fig. 4i), while they were abundantly observed in the wild type ECx. This reduction of inhibitory neurons was also observed at earlier stages (PD20 and PD2 of IER; Fig. 5a–d), suggesting that it is caused by developmental abnormalities rather than defects in adults. Similarly, in *Dscaml1*^*GT2/GT2*^ mice, inhibitory neurons were severely reduced in the ECx (Fig. 5e–h).

### Candidate of *DSCAML1* mutations in epileptic patients

**Fig. 6:**
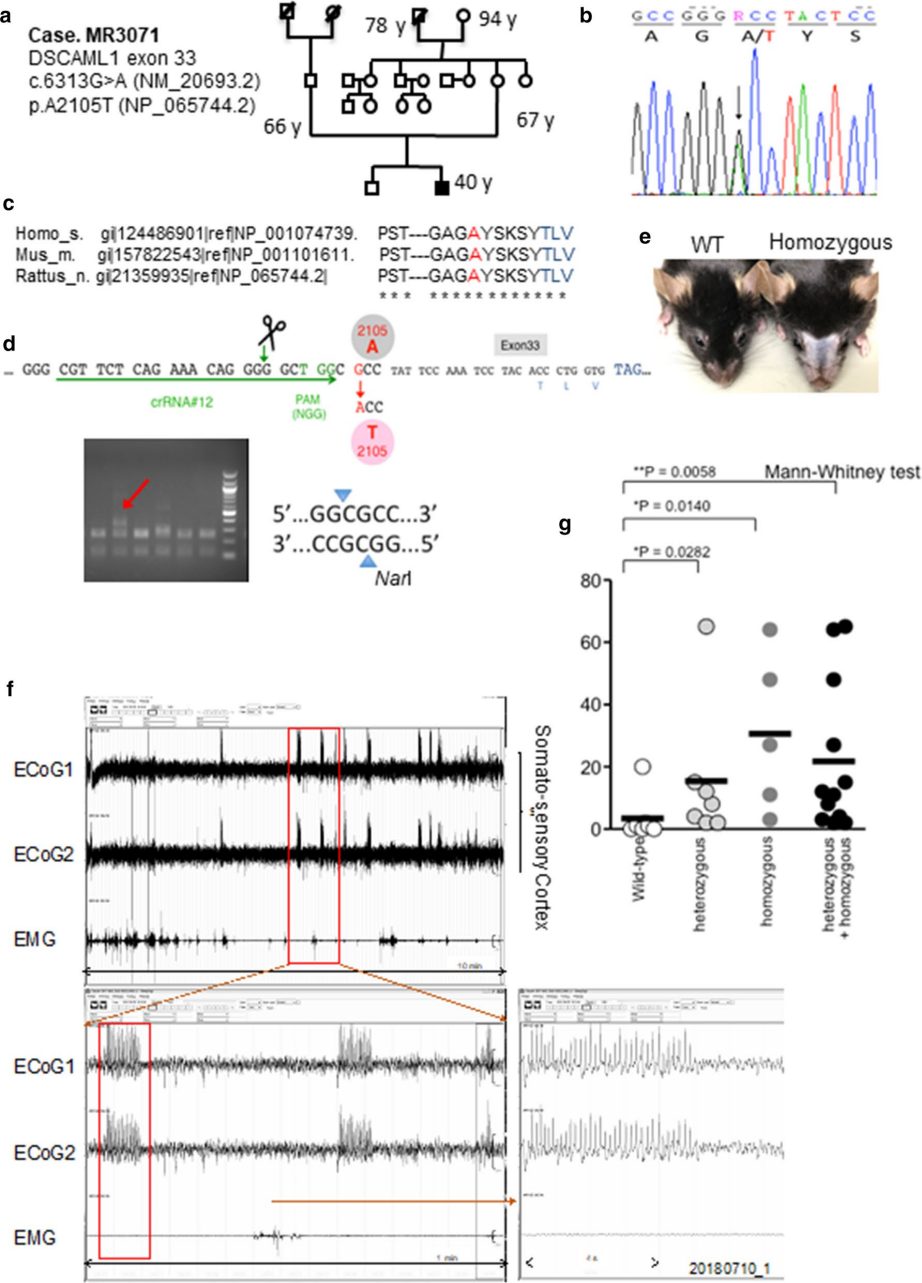
*DSCAML1*^*A2105T*^ mutation of a patient with epilepsy and *Dscaml1*^*A2105T*^ knock-in mice. **a, b** Pedigree and genomic sequence of the *DSCAML1*^*A2105T*^ heterozygous patient. **c** Evolutionarily conserved *c-*terminal protein sequences of DSCAML1. **d** Generation of *Dscaml1*^*A2105T*^ knock-in mice. Negative restriction enzyme (*NarI*) selection of heterozygous founder (red arrow). **e** Scratch-loss of hairs and whiskers in an adult male of *Dscaml1*^*A2105T/A2105T*^ by hyper-grooming. **f** Representative ECoG of *Dscaml1*^A2105T/A2105T^ mouse. **g** Numbers of epileptic spike-and-wave discharges in 6 h (*n* = 5–7, *p < 0.05, **p < 0.01, Man-Whitney U*-*test, Error bars: s.e.m)

Since we identified *Dscaml1* as a novel seizure-related gene in rats, we searched for *DSCAML1* mutations in patients with epilepsy. We performed Sanger sequencing of exons and adjacent sequences of the *DSCAML1* gene in blood samples from the “Depository of the patients with epilepsy and intellectual disability” of our institute under informed consent guidelines [22, 23]. From 30 patients, we found two with *DSCAML1* variants; one is a missense mutation (c.694C > A (NM_200693.2); p.H232N (NP_065744.2)) in exon 3 and the other is a missense mutation (c.6313G > T; p.A2105T) in exon 33 (Fig. 6a, b). In contrast to the allele frequency of the H232N mutation, which is close to 0.5, that of the A2105T mutation is quite low (Additional file 2: Table 2). In addition, the A2105T mutation exhibited high pathogenic potential (Additional file 2: Table 3). Therefore, we focused on the A2105T mutation in this study. The patient, who had average body growth and motor development, carried a heterozygous A2105T mutation and exhibited hyperactivity and autism. He developed epilepsy at age nineteen and was taking anticonvulsant medication. Diagnostic imaging was not performed because of the hyperactivity. His mother was a heterozygous carrier without epileptic seizure. This is similar to IER; IER females rarely show seizures [20]. These results motivated us to test whether the A2105T mutation causes abnormalities that are observed in IER.

### Patient-type *Dscaml1*^*A2105T*^ knock-in mice

The alanine residue at the 2105th position, as well as its surrounding amino acid sequence, is evolutionally highly conserved (Fig. 6c). By CRISPR/Cas9 genome editing [24], we generated *Dscaml1*^*A2105T*^ knock-in mice that carry an amino acid change equivalent to the patient-type DSCAML1 protein (Fig. 6d). DSCAML1 expression levels were not affected in heterozygotes and homozygotes as suggested by immunoblot analysis to hippocampus (data not shown). Although both homozygotes and heterozygotes have no noticeable growth retardation, persistent behaviors such as hyper grooming activity and unconsciousness were observed in both *Dscaml1*^*A2105T*^-genotypes (Fig. 6e, data not shown).

By utilizing the GAD67^GFP^ allele, we found that inhibitory neurons were significantly reduced in the lateral ECx in *Dscaml1*^*A2105T/A2105T*^*; GAD67*^*GFP/*+^ mice, compared to wild type ECx (Fig. 5i, j). This phenotype resembles that of *Dscaml1* loss-of-function mutants, such as IER (Figs. 4i, 5b, d) and Dscaml1^GT2/GT2^ mice (Fig. 5f, h). In ECoG recordings, both heterozygous (*Dscaml1*^*A2105T/*+^) and homozygous (*Dscaml1*^*A2105T/A2105T*^) mice showed abnormal spike-and-waves which were less commonly observed in the WT mice (Fig. 6f, g).

**Fig. 7:**
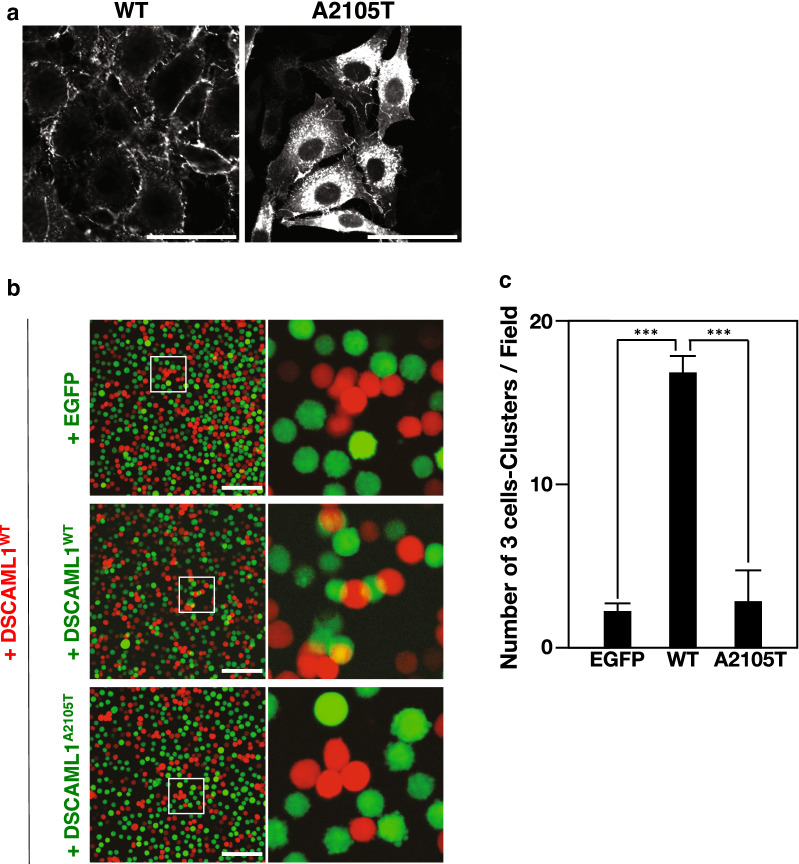
Effects of A2105T mutation on the cellular localization of DSCAML1 and the cell adherences. **a** Fluorescent microscopy images of L cells that stably express DSCAML1^WT^ or DSCAML1^A2105T^. Scale bars: 10 µm. **b** Cell aggregation assay. L cells expressing DSCAML1^WT^ along with mCherry are mixed with L cells expressing EGFP, DSCAML1^WT^ with EGFP, or DSCAML1^A2105T^ with EGFP, respectively. Scale bars: 100 µm. **c** Quantitation of the number of 3 cell-clusters containing red and green cells in the field for each cell line (*n* = 4, ***p < 0.001, Student’s *t*-test, Error bars: s.e.m.)

To analyze the molecular nature of DSCAML1^A2105T^ protein, we generated L929 cell lines (L cell) that stably expressed DSCAML1^WT^ (normal human DSCAML1)-ires-EGFP (or -mCherry), or DSCAML1^A2105T^-ires-EGFP. Surprisingly, while DSCAML1^WT^ was preferentially detected at the cell membrane, as expected, DSCAML1^A2105T^ was mainly observed in the cytoplasm (Fig. 7a). Moreover, in the cell aggregation assays for DSCAML1^A2105T^-expressing cells were unable to adhere to either DSCAML1^A2105T^ or DSCAML1^WT^-expressing cells (Fig. 7b, c), probably due to the lack of DSCAML1 membrane localization. These findings suggest that A2105T-type mutation may disrupt appropriate conformation and subcellular-localization of DSCAML1, leading to loss of function of DSCAML1 and seizure-like phenotypes in *Dscaml1*^*A2105T/A2105T*^ mice.

## Discussion

In this study, we performed linkage studies and genome sequencing and identified *Dscaml1* as the responsible gene for IER, a model rat for human limbic epilepsy. Sequencing analysis identified a single base difference in the genomic-responsible region between IER and ICR (original wild type strain for IER). The one base change from G to A disrupts the consensus sequence of the splicing donor site in exon 12 of *Dscaml1* and results in use of ectopic splicing donor sites and production of erroneous transcripts. The abnormal splicing results in the premature appearance of stop codons and lack of DSCAML1 protein in the IER brain. In addition, expression levels of *Dscaml1* transcripts were extremely reduced in the IER brain. We suspect that the nonsense-mediated RNA decay system may contribute to the reduction of *Dscaml1* transcripts in the IER brains [36], although we lack direct evidence. Extensive human genome and genetic studies have identified a lot of genomic variants in exons and introns which destroy normal splicing of genes and cause a variety of diseases in humans [37]. Therefore, we believe that IER represents a good model for disease-associated splicing mutations.

We observed the clustering of retinal neurons and inhibitory neurons in the IER brains. These phenotypes may be explained by previously reported abnormal neuronal tiling due to loss of self-repulsive function of DSCAML1 [9, 10]. Moreover, we found that GABAergic inhibitory neurons in the outer layers of ECx of the rodent models, such as IER, *Dscaml1*^*GT2/GT2*^, and *Dscaml*^*A2105T/A2105T*^*, *were severely reduced. The insufficient distribution of GABAergic neurons in the ECx may be caused by their dysregulated migration mainly from a ganglionic eminence [38, 39] because the reductions were observed at early developmental stages. However, we do not know how the abnormal distribution of GABAergic neurons take place by the loss of function of *Dscaml1*.

While IER seizure susceptibility is obvious from early postnatal ages, seizures generally appear around PW 25 and gradually increase in frequency and intensity. What induces this transition from the "pre-seizure status" to the "seizure status"? We demonstrated that artificial kindling stimulation at the amygdala at a pre-onset stage easily induced this transition in IER. Moreover, we also showed that electrical stimulation evoked excessive and prolonged neuronal activation in the IER ECx where GABAergic neurons were severely reduced. The layer II neurons in the ECx extend their axons to granule cells in the hippocampal dentate gyrus, through the perforant path [33]. We suspect that natural kindling stimuli [40] are continuously conveyed from the ECx to the dentate gyrus during the pre-symptomatic stages, which eventually brings the IER brain to overt seizures after PW 25.

In the NCNP repository for epilepsy with intellectual disabilities, we identified a heterozygous mutation, *DSCAML1*^*A2105T*^. Protein prediction tools indicated that this A2105T mutation would have a great impact on the three-dimensional structure of DSCAML1 protein. Consistently, the mutant DSCAML1^A2105T^ protein was unable to localize at the cell surface and elicit its cell adhesion function, putatively due to its impaired conformation. We successfully generated a *Dscaml1*^*A2105T*^ knock-in mouse line, mimicking the human mutation. IER and *Dscaml1*^*GT2*^ mouse are loss-of-function mutations and we observed no prominent phenotypes in heterozygotes. However, we observed striking phenotypes even in heterozygous *Dscaml1*^*A2105T*^ mutants. This suggests that DSCAML1^A2105T^ may act as a dominant negative form of wild type DSCAML1 protein, although we do not have direct evidence for this.

Regarding to the A2105T mutation, the patient was a heterozygous male with an asymptomatic mother with the same heterozygous mutation. In IER, almost all males exhibit epileptic seizures by the age of 1 year, while females harbor seizures to a significantly less extent. As gender differences in seizure susceptibility are known [41], we speculated that the mother of the patient did not show symptoms because of the gender difference in seizure susceptibility. However, we do not have any direct evidence and further studies will be required to obtain the mechanistic insights.

From the phenotype of IER and mouse *Dscaml1*^*A2105T*^ and *Dscaml1*^*GT2*^ mutants, it is likely that DSCAML1 mutations cause decreased GABAergic function, at least, in rats and mice that are inbred strains, with very similar genetic background in the same line. This may suggest that loss of function of DSCAML1 is somewhat related to seizure susceptibility in humans. Interestingly, the A2105T variant is present in three healthy heterozygous individuals (two males and one female of African) and in the gnomAD database. In contrast to inbred animals, humans have a variety of genetic background and they are living in different environment. Therefore, we suspect that subtle enhanced seizure susceptibility by DSCAML1 mutations might be hidden in various genetic and environmental background, in addition to gender difference, which results in some humans with DSCAML1 mutations are asymptomatic, while some exhibit seizure-related features. Further studies will be required to assess this issue.

Although we identified *Dscaml1* as the responsible gene for IER, this gene has not been shown to be a causative gene for human epilepsy. However, if it is not a causative gene, it is likely that mutations in this gene affect seizure susceptibility in humans, because *Dscaml1*^*A2105T*^ knock-in mice exhibit spike-and-and-wave ECoG as well as seizure-like behavior.

## Conclusion

We identified *Dscaml1* as the responsible gene for IER. We reseal that a single nucleotide change in IER causes abnormal splicing of *Dscaml1* gene, leading to loss of function of *Dscaml1*. In IER and *Dscaml1* mutant mice, GABAergic neurons were reduced in the ECx, resulting in abnormally enhanced excitability in that region. We found an A2105T-type mutation for DSCAML1 in an epilepsy patient. DSCAML1^A2105T^ lost its normal subcellular localization and cell-adhesion function. Patient-type knock-in mice (*Dscaml1*^*A2105T*^) exhibited phenotypes similar to IER, including seizure-related symptoms. We suspect that *Dscaml1* is related to human seizure susceptibility.

## Supplementary information

**Additional file 1: **Video of abnormal behaviors of IER.

**Additional file 2: **Materials and evaluation of DSCAML1 gene and protein mutation.

## Data Availability

The datasets analyzed during the current study are available from the corresponding author on reasonable request.
