# Supplementary material for: Down syndrome cell adhesion molecule like-1 (DSCAML1) links the GABA system and seizure susceptibility
Source: Acta Neuropathol Commun. 2020 Nov 30;8:206. doi: 10.1186/s40478-020-01082-6 (PMC7706048; doi:10.1186/s40478-020-01082-6)
Supplement: Supplementary file 2 — Additional file 2: Materials and evaluation of DSCAML1 gene and protein mutation. [file 40478_2020_1082_MOESM2_ESM.pdf]

***Down Syndrome Cell Adhesion Molecule Like-1* (*DSCAML1*) links the GABA system and seizure susceptibility**

Authors:

Yoneko Hayase*, Shigeru Amano, Koichi Hashizume, Tadashi Tominaga, Hiroyuki Miyamoto, Yukie Kanno, Yukiko Ueno-Inoue, Takayoshi Inoue, Mayumi Yamada, Shigehiro Ogata, Balan Shabeesh, Ken Hayashi, Yoshiki Miura, Kentaro Tokudome, Yukihiro Ohno, Takuma Nishijo, Toshihiko Momiyama, Yuchio Yanagawa, Akiko Takizawa, Tomoji Mashimo, Tadao Serikawa, Akihiro Sekine, Eiji Nakagawa, Eri Takeshita, Takeo Yoshikawa, Chikako Waga, Ken Inoue, Yu-ichi Goto, Yoichi Nabeshima, Nobuo Ihara, Kazuhiro Yamakawa, Shinichiro Taya*, Mikio Hoshino*.

*Corresponding authors:

Yoneko Hayase MD, PhD, Shinichiro Taya PhD, and Mikio Hoshino MD, PhD

Supplementary material

Supplemental Table 1. List of materials used in this study
Supplemental Table 2. Genotype and minor allele frequency of 2 polymorphisms in the *DSCAML1* gene
Supplemental Table 3. The deleterious prediction of 2 polymorphisms in the DSCAML1 gene by ANNOVAR

Supplemental Table 1. List of materials used in this study

Reagent or resource	Manufacture	Identifier
**Antibodies**		
Rabbit anti-glutaminase (1:500)	Abcam	Cat#ab156876, RRID: AB_2721038
Mouse anti-GAD67 (1:500)	Merck	Cat# MAB5406, RRID: AB_2278725
Rabbit anti-NG2 (1:00)	Merck	Cat# AB5320, RRID: AB_91789
Goat anti-DSCAML1 (1:200)	R&D systems	Cat# AF3315; RRID: AB_2095469
Goat anti-somatostatin (SST) (1:100)	Santa Cruz	Cat# sc-7819, RRID: AB_2302603 (discontinued Dec 31, 2016 due to animal welfare concerns)
Rabbit anti-GFAP (1:500)	Dako Japan	Cat# Z0334, RRID: AB_10013382
Donkey anti- goat, rabbit, mouse and chick fluorescent-conjugated secondary antibodies with Alexa 488, Alexa 568 or Alexa 647	Thermo Fisher	Cat# AlexaR488, AlexaR568, and AlexaR647
**Animals and cell lines**		
ICR/ihr rat (original strain of IER/ihr) (ICR)	Kyoto Univ.	NBRP-rat, #290
IER/ihr rat (IER)	Kyoto Univ.	NBRP-rat, #291
WKY/Izm rat (WKY)	Japan SLC Inc.	WKY/Izm rat
WKAH/Hkm rat (WKAH)	Japan SLC Inc.	WKAH/Hkm rat
C57BL/6N (mouse WT)	Japan SLC Inc.	C57BL/6Nslc
Dscaml1GT2/GT2 (C57BL/6J-Tg) mouse	TIGM, Texas	TG0118 (Dscaml1): AF487347
GAD67GFP KI mouse (CR.Cg-Gad1<tm1.1Tama>)	Gunma Univ.	IMSR Cat# RBRC03674, RRID:IMSR_RBRC03674
DscamlA2105T hDSCAML1 p.2105T A>T KI mouse	This paper	This paper
COS-7 (Cercopithecus aethiops, kidney, SV40 -transformed)	ECA	EC87021302-F0 _ Cell 23:175 (1981)

**Oligonucleotides for the markers of linkage analysis**		
D8Thy1, forward: GTATGATGGGCAGTGAAGAG	NCBI	UniSTS: 120087; 47002972..47003184 Thy1
D8Thy1, reverse: TGTTTGGTCCACAAAGGCTC	NCBI	UniSTS: 120087; 47002972..47003184 Thy1
D8Got62, forward: TTCTAGCTTCTCAGGCACCAA	NCBI	UniSTS: 115842; 47143390..47143613 Cbl
D8Got62, reverse: AGTGGACATCTTACTCAGAGGACA	NCBI	UniSTS: 115842; 47143390..47143613 Cbl
D8Got66, forward: CCACCCTCTCATGGCTCAC	NCBI	UniSTS: 112149; 48458915..48459138 Dscaml1
D8Got66, reverse: AGTGGACATCTTACTCAGAGGACA	NCBI	UniSTS: 112149; 48458915..48459138 Dscaml1
D8Mhos18[a], forward: TTTCCTCATTTGGGAAAGCAC	This paper	NCBI; 48605174..48605624 Dscaml1
D8Mhos18[a], reverse: ACACAAGCCTGTCTCACAC	This paper	NCBI; 48605174..48605624 Dscaml1
D8Mhos11[a], forward: CTAGGCCAAGGAAGCAGATG	This paper	NCBI; 48616352..48616928 Dscaml1
D8Mhos11[a], reverse: GGCAGTAGTCCCCAAAGACA	This paper	NCBI; 48616352..48616928 Dscaml1
D8Mhos9[a], forward: TTTCCTCATTTGGGAAAGCAC	This paper	NCBI; 48640785..48641006 Dscaml1
D8Mhos9[a], reverse: GCTTAGCCTCATTGGTGAGC	This paper	NCBI; 48640785..48641006 Dscaml1
D8Mhos6[a], forward: ATGGATAGGCCTCCTTTTGG	This paper	NCBI; 48653159..48653360 Dscaml1
D8Mhos6[a], reverse: GCCATAATCTGGGTGAGCAT	This paper	NCBI; 48653159..48653360 Dscaml1
D8Mhos3[a], forward: AATCAAAATGGGTGGCTGAG	This paper	NCBI; 48664906..48665056 Dscaml1
D8Mhos3[a], reverse: CCAAAAGCAAGATGGAGAGG	This paper	NCBI; 48664906..48665056 Dscaml1
D8Got69, forward: GAGAGGAGAACTGGAACGGAC	NCBI	UniSTS: 114710; 48836968..48837205 Pcsk7
D8Got69, reverse: TAGGGGACAGCAATTAACAAACA	NCBI	UniSTS: 114710; 48836968..48837205 Pcsk7
D8Arb27, forward: ATGGGATTCATCTGGTTGTAGG	NCBI	UniSTS: 228202; 49151912..49152199 ApoA1
D8Arb27, reverse: CACCTGTTCCAATAGTAGTTGC	NCBI	UniSTS: 228202; 49151912..49152199 ApoA1
**Primer sequence used for the mice genotyping (Dscaml1GT2, Dscaml1A2105T_KI and GAD67_KI)**		
Dscaml1GT2 (LTR-2): AAATGGCGTTACTTAAGCTAGCTTGC	TIGM	TG0118: Gene trapped Cassette (Right)

Dscaml1GT2 (R-TG0118-lower) GACATTGTACGGGCATGACTCAC	TIGM	TG0118: Gene trapped Cassette (Left)
Dscaml1A2105T, forward: GCCACCTTACCTCAGAGG	This paper	
Dscaml1A2105T, reverse: ATTCAGCCACGTTGGTGTG	This paper	
GAD67EGFP, forward: ACGTAAACGGCCACAAGTTC	This paper	Tamamaki, N (Ref. 28)
GAD67EGFP, reverse: GGATCTTGAAGTTCGCCTTG	This paper	Tamamaki, N (Ref. 28)
TaqMan Gene expression assay for rat DSCAML1	Thermo Fisher	Cat# Rn01421925_mH
TaqMan Gene expression assay for mouse Dscaml1	Thermo Fisher	Cat# Mm01174247_m1
TaqMan Gene expression assay for human Dscaml1	Thermo Fisher	Cat# HS0032468_m1
Eukaryotic 18S rRNA endogenous control (VICTM/MGB) probe	Thermo Fisher	Cat# 4319413E
**Primer sequences used for in vitro splicing assay**		
Genomic Dscaml1 containing exon 12–14, forward: AATTGGATCCATGCCAGCCATGATCACGTCGCA	This paper	N/A
Genomic Dscaml1 containing exon 12–14, reverse: AATTGGATCCTCATGATTTGTTCTTGTACTCGA	This paper	N/A
**Primer sequences used for human DSCAML1 genomic sequences**		
Exon 1, forward: CTCCTGTTCCTGGGCTTGT	This paper	N/A
Exon 1, reverse: GGGGCTGACTCTCACTCG	This paper	N/A
Exon 2, forward: AGTCTCTCCCCTTGCCTCTC	This paper	N/A
Exon 2, reverse: ATGCCTATGGACACACAGCA	This paper	N/A
Exon 3, forward: TGAGCTGACTCCCTGTTCCT	This paper	N/A
Exon 3, reverse: TTATCCAGTCCCCACAGAGG	This paper	N/A
Exon 4, forward: AGAGACGCTACCCTGCCTCT	This paper	N/A
Exon 4, reverse: GGTCTTCCTTGGTGGTTTCA	This paper	N/A
Exon 5, forward: TCCCTGAAATGTTCCTCTGG	This paper	N/A

Exon 5, reverse: GGCCTGGTCAGAGACAGGT	This paper	N/A
Exon 6, forward: TACCCCCAGCTACACTCCAC	This paper	N/A
Exon 6, reverse: AGTGAGCGCTGGTTTAATGC	This paper	N/A
Exon 7, forward: GGTTGCGGATAGCAGAAGAA	This paper	N/A
Exon 7, reverse: AACCACAGAGATGGCAAAGG	This paper	N/A
Exon 8, forward: GTAAAAAGGCATCCCTGTGC	This paper	N/A
Exon 8, reverse: TTTTTCTGAATGCCAAGCTG	This paper	N/A
Exon 9, forward: CCAGGGGATCTTTGTTCAAG	This paper	N/A
Exon 9, reverse: CCGTGATTGGAACTGGAAAT	This paper	N/A
Exon 10, forward: GAAGCCCTAGGAGACCCATC	This paper	N/A
Exon 10, reverse: GAGGATGACTCCAGGTGAGG	This paper	N/A
Exon 11, forward: AAATGGCCCTAGATCTGCAA	This paper	N/A
Exon 11, reverse: CCTGCCTCCCCTTCATAGAT	This paper	N/A
Exon 12, forward: GTGGGGTTCCCTGTACTCCT	This paper	N/A
Exon 12, reverse: GGTACATTTTTGGGGTGTGG	This paper	N/A
Exon 13, forward: AAGTCCCTGGGAAGAGAAGC	This paper	N/A
Exon 13, reverse: TTCCTGTCATCCTCCACCAT	This paper	N/A
Exon 14, forward: CTAGGGTGCCAGAGTCCTTG	This paper	N/A
Exon 14, reverse: CCAGTGTCCCTCCCTTCAG	This paper	N/A
Exon 15, forward: GCAGTCTGTGTGAGCTGGAC	This paper	N/A
Exon 15, reverse: TGATCCAGAGGTGGACACAA	This paper	N/A
Exon 16, forward: TTTCAGGGGAACTCAAACAAA	This paper	N/A
Exon 16, reverse: GTGGGGATTATTGTGCTGGA	This paper	N/A
Exon 17, forward: AGCATCATCTCAGCGTGTTG	This paper	N/A

Exon 17, reverse: GTGGACCACAGGACCAAAAT	This paper	N/A
Exon 18, forward: CTAACTGGACGCTCCTCTGG	This paper	N/A
Exon 18, reverse: CGGCCCCTATAGCCATTATC	This paper	N/A
Exon 19, forward: CCTTGAGGTGAAAGACCCTCT	This paper	N/A
Exon 19, reverse: ACAGTTTGAAGGCCAGGAGA	This paper	N/A
Exon 20, forward: ATGGACCCTCCCCTTCTAAA	This paper	N/A
Exon 20, reverse: TGACCCAAGATGGACACTGA	This paper	N/A
Exon 21, forward: ACTCTGTCCGCTGCATCTCT	This paper	N/A
Exon 21, reverse: AGACCCTGTCTGGGGAGAAC	This paper	N/A
Exon 22, forward: GTCAATGTCTCCCAGCCAAT	This paper	N/A
Exon 22, reverse: TGAGAGAGCAGTAGCAGCACA	This paper	N/A
Exon 23, forward: GGCTGGGTTACCTCATACCC	This paper	N/A
Exon 23, reverse: TTCCATTCGATGACCCTCTC	This paper	N/A
Exon 24, forward: GAGAGGGTCATCGAATGGAA	This paper	N/A
Exon 24, reverse: CCCCAGATCGTTTCTGTCAT	This paper	N/A
Exon 25, forward: GACCAAAAGCCTGACTCTGC	This paper	N/A
Exon 25, reverse: AGGCTCCTCCCTTCAGTCTC	This paper	N/A
Exon 26, forward: GCCTCCCACCAAATCTCTCT	This paper	N/A
Exon 26, reverse: TATCTGCCACTCTGCCCACT	This paper	N/A
Exon 27, forward: GAGCAGAGGGGAAGTCCAG	This paper	N/A
Exon 27, reverse: CATCCAGATGGTGCTGTGAG	This paper	N/A
Exon 28-29, forward: ATGCCTCTCCCATTGTCATC	This paper	N/A
Exon 28-29, reverse: GACTTCCATGGGTTGTAGCC	This paper	N/A
Exon 30, forward: TGGGCAACAGAGTGAGACC	This paper	N/A

Exon 30, reverse: CCAGTGCTTTCCATTTTTGC	This paper	N/A
Exon 31, forward: GCTTCTGTGGCTGCATCTG	This paper	N/A
Exon 31, reverse: GAGCTGGTGGGTAGAAGCTG	This paper	N/A
Exon 32, forward: GGGGAAGTGCTCAGTCAGTC	This paper	N/A
Exon 32, reverse: CCTGCTCCTAAGTCCTTGCT	This paper	N/A
Exon 33-1, forward: TGCTTCCAGAGGGCTATGAC	This paper	N/A
Exon 33-1, reverse: CACCAGGGTGTAGGATTTGG	This paper	N/A
Exon 33-2, forward: GGGGTAGGGAGGTCTCAGAA	This paper	N/A
Exon 33-2, reverse: GCATCTGCAAATTGAAGCAA	This paper	N/A
Exon 33-3, forward: CTCGGCTGTTTTTCTGCATT	This paper	N/A
Exon 33-3, reverse: GGGTCGGGCTCTCTCCTC	This paper	N/A
**Reagents used in CRISPR/Cas9 genome editing**		
Guide-it™ Recombinant Cas9	Takara Clontech	Cat# 631442
crRNA: GAGCUAUGCUGUUUUG caggguguaggauuuggaauGUUUUA	FASMAC	N/A
tracrRNA: AAACAGCAUAGCAAGUUAAAAUAAGGCUAGUCCGUUAUCAAC UUGAAAAAGUGGCACCGAGUCGGUGCU	FASMAC	N/A
Single strand DNA containing (c.6307 G>A, mouse Dscaml1): AAACAGCAUAGCAAGUUAAAAUAAGGCUAGUCCGUUAUCAAC UUGAAAAAGUGGCACCGAGUCGGUGCUCACCCCAGGGGTAGGG CGTTCTCAGAAACAGGGGGCTGGCACCTATTCCAAATCCTACAC CCTGGTGTAGGATGGCCGACA	Eurofins genomics	N/A
**Primer sequences used for off-target candidates**		
Chr 4; 144587444 forward: TTCTTCACACGCTTGGTCAG	This paper	N/A
Chr 4; 44587444 reverse: CTATTCCCAAACGCAGAAGC	This paper	N/A
Chr 9; 59380429 forward: TAAAGAAAAAGCCCCCACCT	This paper	N/A
Chr 9; 59380429 forward: TGGCTGAGACCAGTGTTGAC	This paper	N/A

Chr 15; 99249433 forward: CCAGCCCCTTACCTTCTTCT	This paper	N/A
Chr 15; 99249433 reverse: GCCAGACACTGCTGGAAAGT	This paper	N/A
**Chemicals, reagents or resources**		
Medetomidine Hydrochloride	Fuji film-WAKO	Cas RNR:86347-15-1
Midazolam injection 10mg 'Sandoz'	FujiPharma	Cas RNR:59467-70-8
Butorphanol Tartrate	Fuji film-WAKO	Cas RNR:58786-99-5
XylocaineR Jelly 2% (Lidocaine hyderochloride)	Aspen Japan	Cas RNR:137-58-6
Isoflurane anesthesia	Fuji film-WAKO	Cat# 099-06571, CAS# 26675-46-7
pEGFP-C1	Clontech	Cat# 6084-1 (discontinued)
pCAG-IRES2-GFP	Clonthech	Cat# 6029-1 (discontinued)
DSCAML1 (NM_020693) Human Untagged Clone	Origene	Cat# SC304796
Dscaml1 (NM_001081270) Mouse Untagged Clone	Origene	Cat# MC225176
pSV2-neo	ATCC	ATCCR37149TM
BamHI restriction enzyme	NEB	Cat# R0136S
NarI restriction enzyme	NEB	Cat# R0190L
NuSieveTM 3:1 Agarose	Lonza	Cat# 5001
DAPI (1:50000)	Thermo Fisher	Cat# D1306, CAS# 28718-90-3
TO-PROTM-3 Iodoid (642/661) (TOPRO3)	Thermo Fisher	Cat# T3605
Meyer's Hematoxylin stain	Muto, Japan	Cat# 30001
**Equipment and software**		
ABI sequencer	Thermo Fisher	Cat# ABI 3730
CMOS camera	Brainvision Inc., Tokyo	Cat# MiCAM Ultima

Epifluorescence optics	Olympus	Tominaga et al., 2000
MapManager QTX	MapManager	Manly et al. 2001
Leica Application Suite software	Leica	https://www.leica-microsystems.com/
Zen software	Zeiss microscopy	https://www.zeiss.co.jp/microscopy/int/products/microscope-software/zen.html; RRID: SCR_013672
SleepSign for ECoG	Kissei Comtec	https://www.kicnet.co.jp/solutions/biosignal/sleep/eeg_sensor_za/
Prism5	GraphPad	Https://www.graphpad.com/scientific-software /prism/; RRID:SCR_002798

[a] The *D8Mhos3, 6, 9, 11* & *18* markers were newly developed at $(CA)_n$ microsatellite regions between exons 8 & 21 in the *Dscaml1* genome (NC_005107.3 (48363393..48680159) Chr8|Rnor_5.0, NCBI). *D8Mhos3, 6, 9, 11* & *18* are polymorphic between IER and WKHA, and *D8Mhos6, 9, 11* & *18* polymorphic between IER and WKY, respectively.

Supplemental Table 2. Genotype and minor allele frequency of 2 polymorphisms in the *DSCAML1* gene

SNP name	Chromosome (GRCh37)	Nucleotide change	Reference number in dbSNP	Exon	MAF in HGVD[a]	MAF in Tommo[b]	MAF in ExAc[c]	MAF in ExAc (East Asian)
H232N	117403235	c.694C>A	rs3741280	exon4	0.5694	0.558	0.3667	0.5332
A2105T	117299073	c.6313G>A	rs199968404	exon33	0	0	8.3×10^{-6}	0

[a] Human Genetic Variation Disease. [b] Tohoku medical megabank organization. [c] The Exome Aggregation Consortium.

Supplemental Table 3. The deleterious prediction of 2 polymorphisms in the DSCAML1 gene by ANNOVAR

SNP name	SIFT	Polyphen2	MutationTaster	MutationAssessor	PROVEAN
H233N	Tolerated	Benign	Polymorphism automatic	Neutral	Neutral
A2105T	Deleterious	Probably damaging	Disease causing	Low	Neutral
